# Antibody Responses to a Novel *Plasmodium falciparum* Merozoite Surface Protein Vaccine Correlate with Protection against Experimental Malaria Infection in *Aotus* Monkeys

**DOI:** 10.1371/journal.pone.0083704

**Published:** 2014-01-08

**Authors:** David R. Cavanagh, Clemens H. M. Kocken, John H. White, Graeme J. M. Cowan, Kay Samuel, Martin A. Dubbeld, Annemarie Voorberg-van der Wel, Alan W. Thomas, Jana S. McBride, David E. Arnot

**Affiliations:** 1 Institute of Immunology and Infection Research, Center for Immunity, Infection and Evolution, Ashworth Laboratories, University of Edinburgh, Edinburgh, United Kingdom; 2 Biomedical Primate Research Center, Department of Parasitology, Rijswijk, The Netherlands; 3 Scottish National Blood Transfusion Service, Cell Therapy Group, University of Edinburgh, Edinburgh, United Kingdom; University of Copenhagen and Rigshospitalet, Copenhagen, Denmark

## Abstract

The Block 2 region of the merozoite surface protein-1 (MSP-1) of *Plasmodium falciparum* has been identified as a target of protective immunity by a combination of seroepidemiology and parasite population genetics. Immunogenicity studies in small animals and *Aotus* monkeys were used to determine the efficacy of recombinant antigens derived from this region of MSP-1 as a potential vaccine antigen. *Aotus lemurinus griseimembra* monkeys were immunized three times with a recombinant antigen derived from the Block 2 region of MSP-1 of the monkey-adapted challenge strain, FVO of *Plasmodium falciparum*, using an adjuvant suitable for use in humans. Immunofluorescent antibody assays (IFA) against erythrocytes infected with *P. falciparum* using sera from the immunized monkeys showed that the MSP-1 Block 2 antigen induced significant antibody responses to whole malaria parasites. MSP-1 Block 2 antigen-specific enzyme-linked immunosorbent assays (ELISA) showed no significant differences in antibody titers between immunized animals. Immunized animals were challenged with the virulent *P. falciparum* FVO isolate and monitored for 21 days. Two out of four immunized animals were able to control their parasitaemia during the follow-up period, whereas two out of two controls developed fulminating parasitemia. Parasite-specific serum antibody titers measured by IFA were four-fold higher in protected animals than in unprotected animals. In addition, peptide-based epitope mapping of serum antibodies from immunized *Aotus* showed distinct differences in epitope specificities between protected and unprotected animals.

## Introduction

The only malaria vaccine to reach Phase 3 clinical trials (RTS,S) was recently shown to have ∼50% protective efficacy against clinical malaria episodes in 5–17 month old children [1], although protective efficacy waned rapidly and was lost within 3 years [2]. Any fully successful malaria vaccine will require multiple antigenic components derived from multiple parasite lifecycle stages, including antigens from the blood stage, which is often lethal in unprotected, untreated individuals. The erythrocyte invasive stage of *Plasmodium falciparum*, known as the merozoite, is an important target of immune responses which control parasite numbers and hence disease severity [3], [4]. The most abundant protein on the surface of the merozoite is the ∼190 kDa merozoite surface protein 1 (MSP-1). This protein is proteolytically cleaved into a number of fragments during merozoite development and invasion, and antibodies specific for the C-terminal region of MSP-1 have been shown to inhibit this processing [5]. These MSP-1 fragments (p83, p31, p38 and p42) remain as a non-covalent complex on the surface of the merozoite [6] until the p42 component is further cleaved at or just before merozoite invasion [7]. MSP-1 has been the focus of much of the effort to produce an effective vaccine against malaria, and several regions of this major merozoite antigen have been included in vaccine formulations used in both monkeys and humans [8]–[12]. However, little protective efficacy has been elicited in non-human primates without the use of very powerful adjuvants such as Freund's complete adjuvant, which is unsuitable for use in humans and can also have adverse side-effects in the *Aotus* model [13]–[15]. Most vaccine studies on MSP-1 have focused on the conserved C-terminal region of MSP-1, either in the form of MSP-1_42_ or MSP-1_19_ [8], [16]. However, there is evidence that other regions of MSP-1 can elicit functionally protective immune responses. In primate models of malaria, regions of MSP-1 from the N-terminal p83 fragment elicit protective effects *in vivo* [17]. Monoclonal and polyclonal antibodies specific for N-terminal MSP-1 domains have been shown to inhibit parasite growth *in vitro* [18], [19]. The Block 2 region of MSP-1 is a small region of hydrophilic, polar amino acids near the N-terminus of MSP-1, which is highly variable between parasite isolates [20]. Sequence polymorphism in Block 2 is extensive, but all Block 2 sequences can be assigned to three major Block 2 serotypes (K1-like, MAD20-like and RO33-like) named after prototypical parasite strains in which these sequences were first identified [20]–[22]. The K1 and MAD20 Block 2 types contain multiple serine-containing tripeptide repeats, with the general motif ‘SXX’ [20]. The serine residue in the K1 type of Block 2 always uses codon AGT, whereas the codon for S in MAD20 repeats is TCA, presumably to prevent or reduce recombination between these two sequence types [22]. The RO33 Block 2 type contains similar hydrophilic amino acid residues to the other Block 2 types, but is relatively conserved and non-repetitive [20], [22].

We have produced recombinant antigens representing the highly polymorphic and intrinsically unstructured Block 2 region of *Plasmodium falciparum* MSP-1. These proteins are antigenically similar to the native parasite protein and are immunogenic in mice, eliciting antibodies which recognize serotype-specific epitopes within the Block 2 region of the parasite MSP-1 [21]. Human sera from malaria-exposed individuals contain IgG antibodies that recognize very specifically one or another of the three Block 2 serotypes, and correlate with PCR typing of parasites present at the time of infection [23]. Thus, different MSP-1 Block 2 serotypes are immunogenic and antigenically distinguishable when presented during natural infections in humans. In the absence of re-infection, antibody responses to MSP-1 Block 2 decline within a few months of drug treatment and parasite clearance, indicating that naturally induced human antibody responses to Block 2 are short-lived [23]. Human antibody responses to MSP-1 Block 2 are predominantly of the IgG3 subclass [24]–[26], which may explain the short duration of antibody responses to this region, and at least partially explain the requirement for continuous stimulation by malaria infection to maintain clinical immunity to disease in naturally exposed populations [27], [28]. Importantly, and in support of this immunization and challenge trial, we have shown that serum IgG antibodies against the two most frequent allelic types of Block 2 of MSP-1 were strongly associated with protection from *P. falciparum* malaria in Gambian children [29], [30] and in a cohort of children from Ghana over a longer follow-up period [25]. Antibodies to MSP-1 Block 2 are also significantly associated with successful anti-malarial treatment outcomes in children with uncomplicated malaria [31]. The mechanism of action of the antibodies to this polymorphic merozoite antigen have yet to be determined, but probably do not involve invasion-inhibitory effects [32] and may rely on more indirect Fc receptor mediated effects involving innate immune cells [33].

The mechanism(s) of protective immunity to *P. falciparum* in humans are still not fully understood, but may rely at least partly on the acquisition of a network of antibodies to blood stage parasite antigens [3], [4]. Several *in vitro* assays, such as parasite growth inhibition assays (GIA) and antibody dependent cellular inhibition (ADCI) have been developed to test the functional activity of antibodies to parasite antigens, including MSP-1, but none of these assays have yet shown any correlation with clinical efficacy. Although not perfect, non-human primate malaria models for *P. falciparum*, in species such as *Aotus* monkeys, provide an alternative method of *in vivo* assessment of candidate malaria vaccine efficacy [34]–[36], especially where there is no orthologue in any rodent malaria parasite protein, as is the case for MSP-1 Block 2. In this study we show that recombinant Block 2 is immunogenic in mice with a variety of adjuvants suitable for human immunizations. On the basis of these antigen/adjuvant formulation tests, we have tested and validated a recombinant MSP-1 Block 2 vaccine antigen in *Aotus* monkeys by an immunization and virulent parasite challenge trial, using an adjuvant previously used in several human Phase II trials [37].

## Results

### Recombinant antigen production

Since the malaria parasite challenge strain used at BPRC is FVO, recombinant antigens based on the exact MSP-1 Block 2 sequence of the FVO strain were used to produce a homologous immunizing antigen. Genomic DNA from *Aotus* blood containing FVO parasites was used as a template to PCR amplify the Block 2 sequence from the FVO *msp-1* gene. This DNA fragment was cloned into plasmid pGEX-2T as previously described [21], [23]. DNA sequencing confirmed that the challenge strain used at BPRC was identical at the Block 2 locus to the published FVO *msp*-1 sequence [38], and to the Block 2 sequence of the Wellcome isolate [39].

Immunizing antigens were prepared from *E. coli* strains harboring the GST-FVO Block 2 plasmid as described in Materials and Methods. Briefly, the FVO Block 2 antigen was purified in two forms; as a recombinant protein fused to the C-terminus of *S. japonicum* glutathione S- transferase [40] (named GST-FVO) and as a non-tagged FVO Block 2 protein, produced by thrombin cleavage from GST-FVO (named FVO). Details of purification of the two immunogens are given in Materials and Methods.

### Immunogenicity testing of GST-FVO Block 2 and FVO Block 2 recombinant antigens formulated in human-compatible adjuvants

#### ELISA

**Figure 1:**
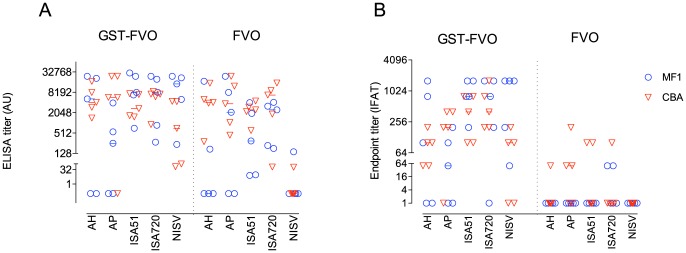
Immunogenicity and adjuvant testing of MSP-1 Block 2 proteins from the FVO isolate. Effect of adjuvants on antibody responses against MSP-1 Block 2. Groups of five outbred (MF1, circles) or inbred (CBA, inverted triangles) mice were immunized s.c. three times at 4 week intervals with FVO MSP-1 Block 2 formulated with the adjuvants as indicated on the X axis. AH, Alhydrogel; AP, AdjuPhos; ISA51, Montanide ISA51; ISA720, Montanide ISA720; NISV, Non-ionic surfactant vesicles. Two weeks after the last immunization, serum samples were tested by ELISA for antibody responses against thrombin-cleaved and purified FVO MSP-1 Block 2 (Panel A) and by IFA reactivity with *P. falciparum* parasites of the Wellcome isolate, which has the same Block 2 sequence as FVO (Panel B). ELISA and IFA titers were calculated as outlined in Materials and Methods. Data is shown on a log_10_ scale as dotplots of serum reactivity for individual animals with the median level of Ab reactivity for each group indicated by a horizontal line.

CBA/Ca or MF1 mice were immunized with either GST-FVO Block 2 or FVO Block 2 recombinant antigens alone, in five different adjuvants suitable for use in humans, namely Alum (Alhydrogel or AdjuPhos), Montanides ISA-51 and ISA-720 and non-ionic surfactant vesicles (NISV). FVO Block 2 specific antibodies were detected by ELISA in the sera of the majority of GST-FVO Block 2 immunized animals 14 days after the third dose, with median titers in each group ranging from 254 (AdjuPhos, MF1 mice) to 14,498 (NISV, MF1 mice) (Figure 1, Panel A, left). Montanides ISA-51 and ISA-720 gave the most consistent antibody titers, with all animals producing significant titers of antibodies above 275, whereas both Alum adjuvant groups had some non-responding animals (Figure 1, Panel A, left). Antibody titers were not significantly lower in the NISV group, but a trend towards a lower median titer was observed.

A larger proportion of non-responding animals were detected in the mice immunized with FVO Block 2 alone (12/50) compared to the four non-responding animals in the GST-FVO immunized groups. Notably, eight of ten animals immunized with FVO Block 2 alone formulated with NISV had no detectable antibodies to FVO Block 2 by ELISA. Four non-responders were observed in the Alum-formulated groups, whereas no mice failed to respond to FVO Block 2 formulated in either Montanide adjuvant. The FVO Block 2/NISV group had significantly lower median antibody titers than several other adjuvant formulations of FVO Block 2 (Alhydrogel, ISA-51 and ISA-720, p<0.05, Kruskal-Wallis test).

#### IFAT

Serum samples from mice immunized with MSP-1 Block 2 were analyzed by IFA for parasite reactivity with the Wellcome isolate of *P. falciparum* by methods previously described [21], [41]. The Wellcome strain was chosen for IFA as it has an identical MSP-1 Block 2 sequence to the FVO isolate.

Mice immunized with GST-FVO Block 2 produced parasite-specific antibodies in combination with all adjuvants tested (Figure 1B, left). All but one animal immunized with GST-FVO Block 2 with both Montanides ISA51 and ISA720 (9/10 mice) had detectable IFA titers to Wellcome parasites. Median (range) titers for the groups were 800 (200–1600) for ISA51/MF1 mice, 400 (100–800) for ISA51/CBA mice, 800 (0–1600) for ISA720/MF1 mice and 400 (200–1600) for ISA720/CBA mice. All four Alum adjuvant groups had lower IFA titers, (median titers 50–200, ranges 0–1600) with sera from 5/20 mice showing no detectable reactivity with parasites. The difference in IFA titers between the Montanide and Alum GST-FVO Block 2 immunized groups was not significantly different (p>0.05, Kruskal-Wallis test), but the higher frequency of non-responders in the Alum groups was notable. GST-FVO Block 2 formulated in NISV elicited IFA titers comparable with the Alum adjuvant groups.

By contrast, mice immunized with FVO Block 2 alone formulated with any of the five adjuvants had low or no parasite-specific antibodies as measured by IFA (Figure 1B, right). Only 9/50 animals had detectable anti-parasite antibodies, with IFA titers ranging from 50 to 200. All other animals had no reactivity with parasites at a 1∶50 dilution of serum. No adjuvant group had more than three responding animals, and median titers were 50 or less in all groups (Figure 1B, right). This contrasted with the FVO Block 2 specific ELISA titers seen in the same sera, which although generally lower than those observed in the GST-FVO immunized groups, did not show such a large discrepancy in antigen-specific titers.

### Immunogenicity of GST-FVO Block 2 in Aotus lemurinus griseimembra

Based on the results of these antigen/adjuvant formulation and immunization studies in mice, *Aotus lemurinus griseimembra* monkeys were immunized with purified GST-FVO Block 2 antigen formulated with Montanide ISA51 as adjuvant (see Table 1 and Materials and Methods for details of protocol and animals). Four animals were immunized three times with GST-FVO Block 2, and three animals were to receive an identical schedule with administration of Montanide ISA51 adjuvant alone (Table 2). One control monkey (A55) died several days after the second immunization date. Necropsy on the animal showed that death was caused by a ruptured heart blood vessel, cause unknown, but not related to experimental procedures. This animal was replaced with a naïve, non-injected animal (A0001) for the parasite challenge stage of the study.

**Table 1:** Details of *Aotus lemurinus griseimembra* used for immunization and challenge.

Group	Number	Sex	Age (years)	Weight (kg)
Experimental	A9801	M	4	0.98
	A9802	M	4	0.92
	A53	F	7	0.87
	A66	M	6	1.02
Control	A9804	M	4	0.73
	A55§ (A0001¶)	M(F¶)	7(2¶)	0.93 (0.90¶)
	A9902*	F	3	0.98
Donor	A31	M	11	0.89

died of non-immunization causes before challenge.

¶naïve (non-injected) animal substitute.

Removed from experiment after challenge.

**Table 2:** Schedule of immunization, blood sampling and parasite challenge of *Aotus lemurinus griseimembra*.

Day (week)	Immunization	Blood sampling	Challenge*
0 (1)	X	X	
14 (2)		X	
28 (4)	X	X	
46 (6)		X	
69 (10)	X	X	
81§ (12)		X	
97 (14)		X	X ¶
120 (17)		X	

The day of sampling and/or immunization is shown with the appropriate week of each time point shown in brackets.

i.v. injection (1 x 10^5^ *P. falciparum* FVO parasites - ring stage).

Parasite challenge Go/No Go decision point, based on IFA titer.

Drug treatment criteria: Parasitemia ≥5% and/or haematocrit ≤20%.

**Figure 2:**
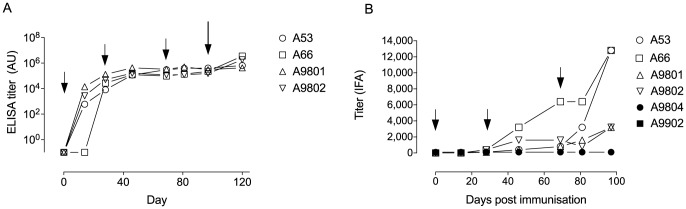
A. Antigen specific antibody titers of sera from *Aotus* monkeys immunized with the GST-FVO Block 2 fusion protein. Serum samples collected at the time points listed in Table 2 were tested for reactivity with cleaved, purified FVO MSP-1 Block 2. Small arrows indicate immunization time points. Large arrow indicates *P. falciparum* challenge time point. Titers were calculated by interpolation from titration curves for each serum sample, with the endpoint titer defined as the dilution that gave an optical density value of 0.1. B. Parasite-reactive antibody titers of sera from four immunized *Aotus* (A53, A66, A9801, A9802) plus control animals (A9804, A9902). Sera were tested by IFA against the Wellcome *P. falciparum* strain (which has an identical MSP-1 Block 2 sequence to FVO). Hollow symbols, immunized animals; filled symbols, control (non-immunized) animals. Small arrows indicate immunization time points.

All four GST-FVO Block 2 immunized animals responded to each immunization by the production of antigen-specific IgG (Figure 2A). ELISA titers in the four animals reached a median level of 38,763 (range 8027 to 127,910) at day 28 after primary immunization, increasing to 207,917 (range 96,769 to 325,618) by day 69 after secondary immunization and 263,692 (range 150,242 to 404,134) by day 97, the day of parasite challenge and 28 days after the 3^rd^ and final immunization (Figure 2A). There was no significant difference in FVO Block 2-specific ELISA titers between immunized animals at any time point (one-way ANOVA, p = 0.1677).

The same serum samples were tested by IFA against fixed Wellcome isolate parasites, which have the same MSP-1 Block 2 sequence as FVO. All four GST-FVO Block 2 immunized animals responded to each immunization by the production of parasite-specific IgG (Figure 2B). IFA titers in the four animals reached a median level of 250 (range 100 to 400) at day 28 after primary immunization, 1200 (range 800 to 6,400) by day 69 after secondary immunization and 8000 (range 3,200 to 12,800) by day 97, the day of parasite challenge (Figure 2B). There was a significant difference in parasite-specific titers between immunized animals at the day 97 time point (one-way ANOVA, p = 0.0312). Day 97 sera from two of the four immunized animals (A53 and A66) had endpoint titers of 12,800; the two other immunized animals (A9801 and A9802) had day 97 titers of 3,200 by IFA (Figure 2B). Control animals had no detectable parasite-specific antibody at any time point. Thus, Ag-specific serum IgG ELISA titers in each immunized animal did not correspond to the parasite-specific IFA titer observed in the same samples (Figures 2A and B).

### Course of parasitaemia and outcomes of infection with FVO parasites

**Figure 3:**
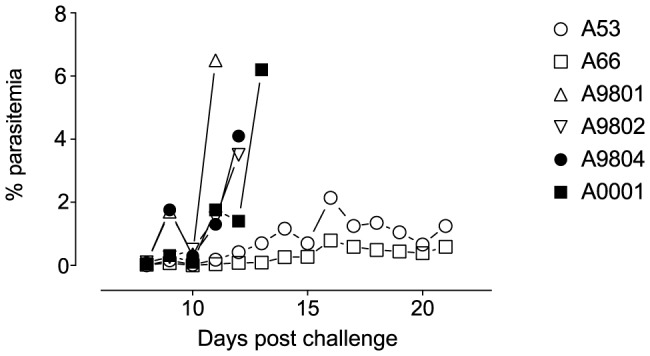
*P. falciparum* parasitaemia development in all challenged *Aotus*. Percentage parasitaemia is plotted against day of sampling post challenge. Open symbols, immunized animals; filled symbols, control (naïve, non-immunized) animals.

The protective efficacy of antibodies induced by GST-FVO immunization, compared with administration of adjuvant alone or in naïve controls was assessed in *Aotus lemurinus griseimembra* monkeys. Animals were infected with 10^5^ FVO ring stage parasites from a donor monkey (A31). One control monkey (A9902) died for unknown reasons shortly after parasite challenge. All other animals became patent for parasites (i.e. positive blood film) on day 8, apart from A53, which became patent on day 9 (Figure 3). Two of the four GST-FVO Block 2 immunized animals were not protected, and were treated with mefloquine on days 11 (A9801) and 12 (A9802) for parasitemias above or approaching 5%. The two remaining negative control animals were also treated with mefloquine on days 12 (A9804, adjuvant control) and day 13 (A0001, naïve control) for the same reason. The two remaining immunized animals (A53 and A66) were defined in this study as protected from severe infection, i.e. were able to control parasitaemia below 2.2% throughout the 21-day follow-up period (Figure 3). One animal (A66) controlled parasite levels below 0.8% throughout this time, whereas A53 reached a peak parasitaemia of 2.14% on day 16, then maintaining parasite levels at ∼1% until the end of the trial. These two animals were then treated with mefloquine on day 21 as agreed in the trial protocol. No animal became anemic during this trial.

### Fine-scale epitope mapping of antibody responses to GST-FVO Block 2 in immunized Aotus

**Figure 4:**
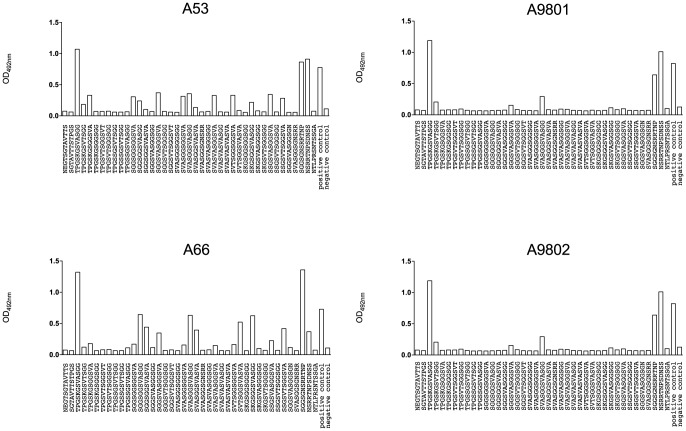
Recognition of peptide epitopes within MSP-1 Block 2. A panel of 38 N-terminally biotinylated dodecapeptides representing the sequence diversity of the MSP-1 MAD20 Block 2 serotype was used in ELISA to map the antibody specificities present in the sera of immunized monkeys, as described in Materials and Methods. Reactivity with individual peptides is shown in columns, with the strength of reactivity of each serum sample with each peptide shown as ELISA optical density. The sequence of each peptide used is indicated on the X-axis below each column.

**Figure 5:**
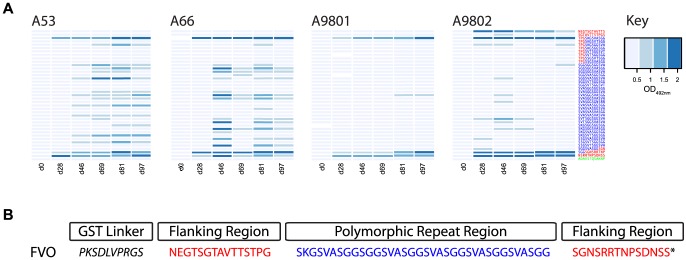
A. Heat map of antibody reactivity to FVO Block 2 serotype peptides over the course of immunization. MSP-1 Block 2 specific peptide ELISA is as described in Figure 4 and in Materials and Methods. Reactivities of sera from immunized *Aotus* are shown as blue rectangles for each peptide tested, with darker colored bars indicating higher ELISA reactivity as shown in the figure key. Columns represent serum reactivity for each time point, and each panel shows reactivity for all pre-challenge samples from each animal. B. Amino acid sequence of the FVO MSP-1 Block 2 antigen. MSP-1 Block 2 flanking sequences are shown in red and internal repeat sequences in blue, matching the peptide sequences shown in Panel A.

A panel of 38 N-terminal biotinylated 12-mer peptides, representative of all major linear epitopes in the homologous Block 2 serotype of MSP-1 were used to fine-scale map antibody responses in all immunized animals. This peptide panel included all known repetitive sequences from the MAD20 Block 2 serotype, and peptides from the non-repetitive flanking regions of the MAD20 Block 2 serotype. Analysis of day 97 sera from the four animals showed a striking difference in epitope specificity between the two protected monkeys that controlled infection (A53 and A66, which had the highest IFA titers) and the two non-protected animals (A9801 and A9802, which had four-fold lower IFA titers). Sera from protected animals A53 and A66 both contained antibodies specific for the repetitive amino acid sequences in the central part of the FVO Block 2 sequence (Figures 4A and 4C and Figure 5). The two non-protected animals had very low levels of these antibodies, and a very narrow range of peptide specificity, recognizing only one peptide weakly [SVASGGSVASGG] (Figure 4B and 4D). Serum from all animals had detectable antibodies to the non-repetitive flanking sequences of the FVO Block 2 antigen, which were of similar intensity in all four samples (Figure 4, all panels). This selective recognition of Block 2 peptide epitopes has been observed in mice immunized with MSP-1 Block 2 antigens, especially when formulated with adjuvants less powerful than Freund's complete adjuvant [32], [42]. Thus the main difference between protected and non-protected animals appears to be a broader epitope specificity of antibodies in the protected animals, which is reflected in the higher IFA titers in the two protected monkeys. The development of these responses for all animals over the immunization period is shown as a heat map in Figure 5. Antibodies to the flanking regions of FVO Block 2 appeared after the first immunization and remained high in all four animals. By contrast, repeat sequence specific antibodies were not detectable until after the second immunization in three animals (day 46, Figure 5). In the two protected animals, A53 and A66, these antibodies were maintained or enhanced in all subsequent serum samples, whereas the two non-protected animals either failed to maintain the weak reactivity initially detected (A9802, Figure 5) or failed to produce any significant levels of antibody to any repeat sequence epitope (A9801, Figure 5).

### Parasite–specific antibodies in Aotus sera, pre- and post-challenge

**Figure 6:**
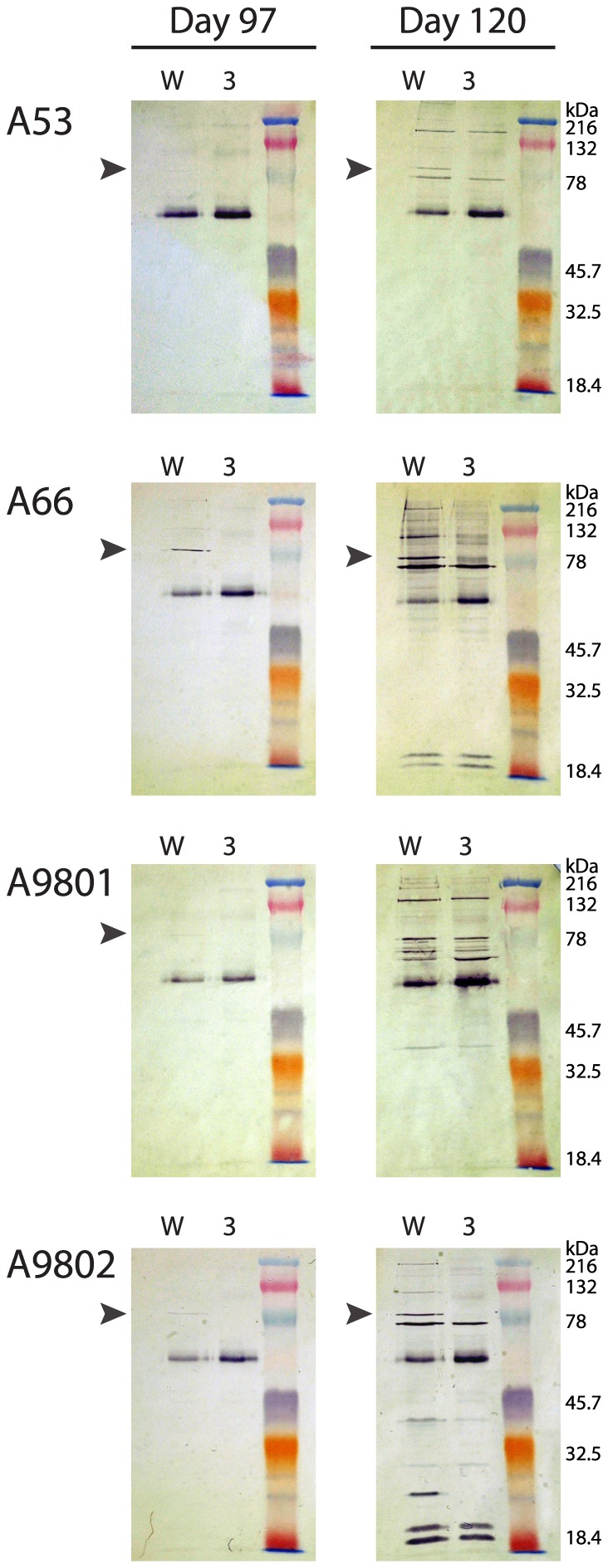
Reactivity of *Aotus* sera with parasite proteins. Schizont extracts from the Wellcome (W) and 3D7 (3) isolates were probed by Western blotting with sera from all four immunized animals. Serum samples from day 97 (pre-challenge) and day 120 (post challenge) from each animal were tested in parallel on contiguous parts of the same membrane. Immunized animal code numbers are shown on the left of each panel. Arrowheads indicate reactivity with the N-terminal p83 proteolytic fragment of MSP-1. The dominant 50 kDa band in all blots is the heavy chain of human IgG, recognized by the secondary reagent (HRP conjugated anti-human IgG heavy chain).

Serum samples from each monkey on day 97 (day 0 of challenge) and day 120 (23 days post-challenge) were used to detect parasite proteins from schizont extracts by Western blotting, using proteins extracted from Wellcome (Block 2 homologous) and 3D7 (Block 2 non-homologous) parasites. In all four animals it was possible to detect antibodies reacting with the N-terminal p83 fragment of mature Wellcome MSP-1, which contains the Block 2 region [6], [43], [44] (Figure 6, arrowed). The strongest pre-challenge reactivity was seen in monkey A66, where a distinct band specific for schizont MSP-1 from the Wellcome isolate (but not MSP-1 from 3D7 parasites) was clearly visible (Figure 6, A66 left side, arrowed). All other reactivities in the day 97 samples, including the strong reactivity with a ∼50 kDa protein seen in all lanes, are caused by reactivity of the secondary HRP-conjugated anti-human IgG antibody used with human IgG present in the parasite extracts (Figure 6, all panels, left side).

In three out of four animals, there is a distinct, strain-specific boost in reactivity to the p83 component of MSP-1, except for A9801, where reactivity with the MSP-1 p83 component is undetectable in the day 120 sample, despite reactivity at day 97 (Figure 6, A9801, right side). A9801 was not protected after challenge; the failure of the immunization to protect may therefore be related to the lack of parasite-specific antibody boosting seen in this animal. By contrast, animal A9802, which was also not protected after challenge, showed a distinct, strain-specific boost in reactivity against the homologous Wellcome p83 fragment of MSP-1 23 days after challenge. This strain-specific but non-protective response may reflect the 24-hour delay in the rise in parasitemia seen in animal A9802 compared to the earlier rise in parasitemia seen in animal A9801 (Figure 3).

## Discussion

We have demonstrated in this study that *Aotus* monkeys can be partially protected against a virulent, normally lethal challenge with the FVO isolate of *P. falciparum*, by immunization with GST-FVO MSP-1 Block 2 antigen. Importantly, protective titers of antigen-specific antibody can be elicited using an adjuvant already in use in humans, Montanide ISA51, which has been used in more than 25 vaccine clinical trials with acceptable levels of reactogenicity [45]. To our knowledge, very few malaria vaccine trials in *Aotus* monkeys have successfully protected animals from blood stage challenge using adjuvants acceptable for humans [46], [47] and most studies using such adjuvants have failed to provide any level of protection using the highly virulent *P. falciparum* isolate FVO challenge model [11], [47], [48]. A much larger number of trials have been conducted using Complete Freund's adjuvant, with varying degrees of success in conferring protection in several *Aotus* models [10], [17], [49]–[53]

More importantly, we have also shown that parasite-specific antibody titers, as determined by IFA, correlate closely with protective immunity to parasite challenge. Immunization of *Aotus* monkeys with GST-FVO Block 2 induced antibodies to the flanking sequences of the Block 2 antigen in all animals, but only two animals also had antibodies to the internal repetitive epitopes of Block 2 and were protected from parasite challenge. The association between the presence of repeat-specific antibodies and protection in this immunization and challenge trial is supported by the observation that antibodies to repeat-specific epitopes within MSP-1 Block 2 are associated with reduced risk of clinical malaria episodes in cohorts of naturally exposed African children [25], [30]. Evidence from this study indicates that the development of Block 2 repeat sequence-specific antibodies only occurs after repeated immunization with the GST-FVO Block 2 antigen, as the two protected animals only developed detectable antibodies to the repeat sequences after two or three immunizations (Figure 5, monkeys A53 and A66).

The reason that antibodies to the repetitive amino acid sequences are almost undetectable in the immunized but non-protected animals (A9801 and A9802) is unclear, but may reflect the outbred nature of the monkeys used, or differences in ages of the animals (A9801 and A9802 were 4 years old, A53 and A66 were 7 and 6 years old), although all animals were adults (Table 1). In naturally exposed humans, the need for the acquisition of multiple antibody specificities to a network of antigen and epitope specificities is thought to be one reason for the relatively slow development of immunity to malaria [3], [4]. It may be that the intrinsic immunogenicity of disordered protein sequences is lower than that of more conserved regions of MSP-1, such as the flanking sequences of Block 2 [54] or conserved globular domains such as MSP-1_19_, which are more frequently encountered during natural infections [23].

Promisingly, measurement of specific antibodies by IFA, ELISA, Western blotting and peptide epitope mapping confirmed that the responses to MSP-1 Block 2 induced by immunization were boosted by challenge with *P. falciparum* (Figures 2 and 6). This boosting of antibody responses by infection has significant practical importance, as this confirms that the GST-FVO Block 2 antigen has sufficient immunological identity to the parasite MSP-1 Block 2 region to stimulate appropriate and specific B cell memory in the immunized animals.

In this trial, immunized animals were challenged with 1×10^5^ FVO ring stage parasites. Most recent studies using the highly virulent *P. falciparum* strain FVO have challenged with parasite numbers ranging from 1×10^4^ [11], [13], [47], [55] to 5×10^4^ [10], [56], [57] and occasionally 1×10^5^ [58]; thus we have used an inoculum at the highest end of the range used in other studies. In addition, *Aotus lemurinus griseimembra* monkeys are considered to be the most susceptible species to infection with *P. falciparum* asexual blood stages [59]. Montanide ISA51 has not been used as the sole adjuvant in any other *Aotus* trial to our knowledge, having been combined with Complete Freund's adjuvant in all previously published studies. Thus the combination of a 2-10 fold higher parasite inoculum, the use of a human acceptable adjuvant such as Montanide ISA51 and the highly susceptible species *A. lemurinus griseimembra* indicates that this trial, although using only small numbers of animals, has shown a highly significant protective effect which is convincingly explained by very specific antibody responses to defined epitopes within MSP-1 Block 2.

We conclude that immunization with a Block 2 antigen derived from MSP-1 induces strongly protective immune responses, albeit not in all immunized monkeys. Using close monitoring of the antibody responses induced by vaccination, including fine-scale epitope mapping of the response to immunization, we have further validated the Block 2 region of MSP-1 as a promising malaria vaccine candidate. Thus, our data are fully in line with the earlier observations that protection from malaria in the field strongly correlates with the presence of Block 2 antibodies [25], [29], [30], [32].

The mechanism(s) by which antibodies to Block 2 act to inhibit parasite growth and/or development in the host are not understood, but like antibodies to other merozoite antigens (e.g. MSP-2, MSP-3), they may have anti-parasite effects via Fc receptor-mediated activation of innate immune cells, as measured by assays such as antibody dependent cellular inhibition (ADCI) [33] or antibody dependent respiratory burst [60]. Since MSP-1 Block 2 is highly polymorphic, in endemic settings acquiring a repertoire of antibodies to repeat sequence variants from each MSP-1 Block 2 type would require repeated infections with parasite isolates presenting each of the three Block 2 serotypes. An effective vaccine based on a polymorphic protein domain such as MSP-1 Block 2 would therefore need to elicit antibodies to all three MSP-1 Block 2 serotypes, including sequence and antigenic variants within each type. This is an achievable requirement, as we have now constructed and successfully pre-clinically tested such a vaccine antigen [32].

The limited number of blood-stage vaccines that have reached clinical trials have shown disappointing levels of efficacy, with only three candidates reaching phase 2b studies [61]. Merozoite antigens are often polymorphic, and the monomorphic merozoite protein vaccines trialled thus far have shown, unsurprisingly, allele-specific signatures of protection [62]–[64]. We identified this polymorphic antigen region from MSP-1 that showed population-level signatures of immune selection [29], and have been able to produce an immunogenic vaccine antigen which can elicit antibodies to diverse allelic types of MSP-1 [32]. It is now possible to develop a vaccine based on MSP-1 Block 2 that is designed to protect against all parasite genotypes and resist immune escape. Such a diversity covering blood stage vaccine, combined with an effective pre-erythrocytic antigen would be an attractive option.

## Materials and Methods

### Animal welfare and ethical clearance

Aotus monkeys (*Aotus lemurinus griseimembra*) used in this study were captive bred for research purposes. They were socially housed at the BPRC facilities in cages of 150×75×185 cm, under compliance with the Dutch law on animal experiments, European directive 86/609/EEC and with the ‘Standard for humane care and use of Laboratory Animals by Foreign institutions’ identification number A5539-01, provided by the Department of Health and Human Services of the USA National Institutes of Health (NIH). To minimize distress to the animals they were housed under a reversed day/night rhythm so that animal handling occurred when the animals were awake. Nonhuman primates were used because no other models (*in vitro* or *in vivo*) were available for testing of experimental human malaria vaccines. Standard feeding regime consisted of Marex food for New World monkeys, water ad libitum and food enrichment in the form of fruit chains (whole fruit, apples, bananas and vegetables, peppers, endive etc, on a chain). Cage enrichment was present in the form of ropes, branches, elevated seating plateau and shelter cages. All animals were daily monitored for health and discomfort. The local independent ethical committee constituted conform Dutch law (BPRC Dier Experimenten Commissie, DEC), approved all research protocols prior to the start. The composition of the DEC and the decision process are based on the Dutch “Experiments on Animals Act” (Wet op de Dierproeven) and the “Experiments on Animals Studies Decision” (Dierproevenbesluit). Both documents are available online on http://wetten.overheid.nl. All experiments were performed according to Dutch and European laws. The Council of the Association for Assessment and Accreditation of Laboratory Animal Care (AAALAC International) has awarded BPRC full accreditation. Thus, BPRC is fully compliant with the international demands on animal studies and welfare as set forth by the European Convention for the Protection of Vertebrate Animals used for Experimental and other Scientific Purposes, Council of Europe (ETS 123), Dutch implementing legislation and the Guide for Care and Use of Laboratory Animals. At the end of the experiment all monkeys were radically cured with an oral dosage of mefloquine, subsequently monitored for absence of blood stage parasites and then returned to the colony. All intravenous injections and large blood collections were performed under ketamine sedation, and all efforts were made to minimize suffering.

### MSP-1 FVO Block 2 construct

DNA was obtained from *Aotus* peripheral blood containing the *P. falciparum* isolate FVO, which is used as the challenge strain for *Aotus* monkeys at the Biomedical Primate Research center, Rijswijk, the Netherlands. Parasite DNA was purified using the method of Fenton *et al.* [65] and used as a template for an MSP-1 Block 2 specific PCR reaction. Each reaction contained 10 mM Tris-HCl (pH 8.3), 50 mM KCl, 1.5 mM MgCl_2_, 0.001% (w/v) gelatin, 2 mM each dNTP, 2.5 units Taq DNA polymerase (Roche Diagnostics Ltd., UK) and 1 mM each of the following pairs of primers: forward primer (5′ CTGGATCC AATGAAGGAACAAGTGGA 3′) and reverse primer (5′ GGGAATTCTTA ACTTGAATTATCTGAAGG 3′). Underlined portions of each primer indicate non-MSP-1 sequences added to incorporate *Bam*HI and *Eco*RI restriction enzyme sites into the PCR products, whereas non-underlined parts of these primers match specifically the 5′ and 3′ ends of the MAD20 type Block 2 sequence of MSP-1. A polymerase chain reaction (PCR) cycle of 95°C, 90 s; 50°C, 15 s; 72°C, 45 s was repeated for 35 cycles in each case.

### Production of recombinant MSP-1 FVO Block 2 antigens

The PCR amplified Block 2 region of the FVO MSP-1 gene was expressed in *E. coli* as a recombinant protein fused to the C-terminus of glutathione S-transferase (GST) of *Schistosoma japonicum* using the pGEX-2T vector [40]. This protein, known as GST-FVO BL2, was produced and purified from cultures of *E.coli* harboring the GST-FVO BL2 plasmid construct. The GST-FVO BL2 fusion protein was purified to homogeneity by affinity chromatography on a Hi-Trap GST column using the standard manufacturer's protocol (GE Healthcare, UK). Cleaved FVO protein was produced by digestion of GST-FVOBL2 with thrombin protease (Amersham) following the manufacturer's protocol. Thrombin and cleaved GST were removed from the digested GST-FVO fusion protein by affinity chromatography of the digested material on a Hi-Trap GST column, followed by ultrafiltration (repeated five times) through a Vivaspin 20,000 M_r_ cutoff centrifugal filter (Vivascience Inc., USA). The filtrate contained the 5.3 kDa FVO Block 2 protein alone. Purified proteins were dialyzed extensively against PBS, filter sterilized and stored at −70°C until needed.

### Immunogenicity testing

Purified GST fusion proteins were used as immunogens to produce polyclonal antisera in inbred (CBA/Ca) and outbred (MF1) strains of mice. Immunizing antigen was prepared by diluting each purified protein in PBS (7.15 mM Na_2_HPO_4_, 2.85 mM KH_2_PO_4_, 3.58 mM KCl, 0.134 M NaCl), and formulating in a series of five adjuvants: Alhydrogel (AlOH_3_) or AdjuPhos (AlPO_4_) (Brenntag Biosector, Denmark); Montanide ISA51 or Montanide ISA720 (Seppic, France) or non-ionic surfactant vesicles (NISV) [66]. These mixtures were used to immunize mice intraperitoneally. For each mouse, 50 µg of protein in a final volume of 300 µl was used for each immunizing dose. Three doses were given at monthly intervals and blood was collected 12–14 days after the final dose. Sera from blood samples were tested by IFA and ELISA for parasite- and antigen- specific titers.

### Indirect Immunofluorescence Assays (IFA)

Specificities and titers of mouse and *Aotus* antisera elicited by immunization with the recombinant FVO Block 2 proteins were compared by indirect immunofluorescence assays (IFA) on fixed parasite preparations as described (2;6). Serial dilutions of the antisera (1∶50 – 1∶ 51,200), or control monoclonal antibodies, were made in PBS containing 1% BSA and 0.01% sodium azide. A 25 µl volume of each antiserum was incubated on a well of a multispot slide (Hendley, Essex, UK) with acetone-fixed schizonts at room temperature for 30 min, followed by washing of the slides in PBS (1 min/5 min/5 min). The slides were dried for 10 min at 50°C, then each spot was incubated with 15 µl of 1∶80 dilution of FITC (fluorescein isothiocyanate) conjugated rabbit anti-mouse Ig (ICN) for 30 min at room temperature. After two washes in PBS, the slides were immersed in a solution of 0.1% (w/v) Evans Blue and 0.001% (w/v) DAPI (4′,6-diamino-2- phenylindole, Sigma) in PBS for 5 min to counter stain erythrocytes and parasite nuclei respectively. The slides were washed and mounted in Citifluor (City University, London) under cover-slips. The parasites were visualized by DAPI-fluorescence (DNA specific) and antibody-reactive parasites by FITC-fluorescence (Ab specific) with incident light of 450–490 nm and 390–440 nm respectively, at a magnification of x 600.

### Immunization and challenge protocol for Aotus monkeys

Eight adult *Aotus lemurinus griseimembra* monkeys were selected from a group of eleven animals, on the basis of non-reactivity with *P. falciparum* parasites, as measured by IFA at the Biomedical Primate Research Centre (BPRC), Rijswijk, Netherlands and by non-reactivity in an MSP-1 Block 2 ELISA in Edinburgh, using serum from routine health checks. One animal was reserved as parasite donor for the other seven, in the event of parasite challenge proceeding. The other seven animals were assigned to one group of four and one group of three monkeys, with age, weight and sex matched. A summary of the animals used is given in Table 1.

Four animals (A9801, A9802, A53, A66) were immunized with 100 µg of GST-FVO Block 2 formulated in Montanide ISA51, prepared according to the manufacturer's protocol (Seppic, Paris, France). Three control animals (A9804, A55, A9902) were immunized with PBS in Montanide ISA51. Animals were immunized by i.m. injection three times with the same dose on days 1, 28 and 69. Blood samples (1–3 ml) were taken from each animal on days 1, 14, 28, 46, 69, 81, 97, and 120. Sera collected from animals on days 1, 14, 28 and 46 were sent together to Edinburgh for analysis by ELISA and IFA, to check for anti-Block 2 and anti-parasite antibody titers respectively. Samples were sent “blinded” to the Edinburgh investigators to prevent bias. In a similar manner, sera collected on day 81 (and from day 69) were sent to Edinburgh for IFA and ELISA analysis by day 84. The schedule of immunization and sampling is summarized in Table 2.

Seroconversion of the immunized animals was confirmed on day 84, as agreed in a prior immunization and challenge protocol devised in advance of the study by the investigators and the veterinarians at BPRC. We used the *P. falciparum* Vietnam Oak Knoll (FVO) strain, which is adapted to growth in *Aotus* monkeys. FVO intravenous infection has been shown to produce a reproducible, lethal, high-density parasitemia in naive monkeys with intact spleens [8], [16], [67]. A stock inoculum of FVO was thawed on day 84 and injected into the donor animal (1×10^5^ parasites) on day 85. The animal was monitored for parasitaemia by microscopy of Giemsa-stained blood smears from day 88 onwards, until a parasitaemia of 0.5-1% was reached. A blood sample of 5 ml was taken and used to challenge all seven Aotus monkeys by i.v. injection (1×10^5^ parasites in total volume of 0.5 ml). Injections were given to control animals first and last, and all other animals were randomised in between these.

The general health of the animals was monitored daily and noted in the study book. Parasitaemia in each animal was monitored daily from day 97 onwards (blinded) by microscopic examination of Giemsa-stained thin films prepared from a small drop of blood from a needle prick in the monkey calf, from hand-held monkeys. Monkeys were given a reward following this procedure. If low parasitemia was observed for several days, haematocrit was checked daily in addition to parasitaemia. Animals were treated orally with 30 mg mefloquine in 3 ml 0.9% NaCl if parasitaemia reached ≥5% and/or haematocrit fell to ≤25%. Treated monkeys were monitored several times by microscopy of Giemsa-stained thin blood smears to verify clearance of parasites. A final blood sample was taken from all animals on day 23-post challenge.

### Western blotting

Schizont proteins extracted in SDS-sample buffer (50 mM Tris pH 6.8, 5% [v/v] 2-mercaptoethanol, 2% [w/v] SDS, 0.1% [w/v] bromophenol blue, 10% [v/v] glycerol) were resolved by discontinuous SDS-PAGE in 10% acrylamide gels and transferred to Protran BA85 membranes (Schleicher and Schuell) in a MiniProtean III electrophoresis unit (BioRad, UK) at a constant current of 90 mA with cooling for 1 h. The membranes were incubated in blocking buffer (5% non-fat milk powder in PBS, supplemented with 0.05% Tween 20 and 0.02% NaN_3_) for 1 h, and the transferred proteins then probed for 3 h with *Aotus* serum samples from day 97 (parasite challenge day 0), and from day 120 (23 days post challenge) diluted 1∶500 in blocking buffer. The membranes were washed three times for 10 min each in washing buffer (0.05% (v/v) Tween 20 in PBS), incubated with horseradish peroxidase-conjugated rabbit anti-human IgG antibody (Dako, UK) diluted 1∶500 in washing buffer for 1 hour, washed three times as above and finally rinsed in 10 mM Tris, 0.9% NaCl, pH 7.4. Binding of mouse IgG to schizont proteins was visualised using H_2_O_2_ and 4-chloro-1-naphthol as the chromogenic substrate (5).

### Enzyme-linked Immunosorbent Assay (ELISA)

*Aotus* sera were tested by ELISA for their ability to recognise the recombinant MSP-1 fragments from Block 2. Wells of 96-well plates (Immulon 4 HBX, Dynex) were coated with 50 ng/well of recombinant antigens in 100 µl of coating buffer (15 mM Na_2_CO_3_, 35 mM NaHCO_3_, pH 9.3) overnight at 4°C. The wells were washed three times in washing buffer (0.05% Tween 20 in PBS). Unoccupied protein binding sites were blocked with 200 µl/well blocking buffer (1% [w/v] skimmed milk powder in washing buffer) for 5 h at room temperature and washed again three times. Mouse or *Aotus* sera diluted in the blocking buffer (100 µl per well) were added to duplicate antigen-coated wells overnight at 4°C. After three washes, the wells were incubated for 3 h with 100 µl per well of horseradish peroxidase-conjugated polyclonal rabbit anti-mouse IgG (at 1∶1000) or rabbit anti-human IgG (at 1∶5000) (Dako Ltd., High Wycombe, UK) and washed three times before incubating for up to 15 min at room temperature with 100 µl of substrate (0.1 mgml^−1^ o-phenylenediamine [Sigma] and 0.012% H_2_O_2_) in development buffer (24.5 mM citric acid monohydrate and 52 mM Na_2_HPO_4_, pH 5.0). The reaction was stopped with 20 µl of 2 M H_2_SO_4_ and absorbance was measured at 492 nm. Endpoint titres were calculated by interpolation from titration curves for each serum sample, with the endpoint titer defined as the dilution that gave an optical density value of 0.1 at 492 nm.

### Epitope mapping by biotinylated peptide ELISA

A set of 38 biotinylated dodecapeptides covering all possible linear epitopes contained within MSP-1 MAD20 Block 2 type sequences were synthesized by Mimotopes Pyt. Ltd. (Clayton, Australia). ELISA plates (Immulon 4 HBX, Thermo Dynex) were coated with 100 µL of 5 µg ml^−1^ streptavidin (Sigma) and incubated at 37°C until dry. Plates were stored in heat sealed foil pouches with 1 g silica gel at room temperature until use. Reactivity of sera against the peptide library was determined by ELISA. Streptavidin-coated plates were washed in PBS-T (PBS/0.05% Tween20) and blocked with blocking buffer (1% ByCoA, Croda Healthcare, UK dissolved in PBS) for 5 hours at room temperature. Peptide library plates were prepared by addition of 300 ng peptide per well, in duplicate, and plates were incubated overnight at 4°C. Sera were added to each well (100 µL at 1∶500 dilution) and incubated overnight at 4°C, then washed with PBS-T. Dilutions of a species-specific HRP-linked secondary antibody (Dako, UK), appropriate to the serum being tested, were added to each well and plates were incubated at room temperature for 3 hours. Plates were washed three times with PBS-T and OPD substrate was added to each well. Reactions were stopped by addition of sulfuric acid and absorbance was read at 492 nm using a microplate absorbance reader (Multiskan Ascent, Thermo Scientific, UK). Background reactivity was calculated as the mean of all OD values in the lowest two quartiles (i.e. below median)+6 standard deviations. Peptide reactivity data was then categorized into the following groups; high  =  background +1 OD, medium  =  background +0.5 OD, low  =  greater than background, negative  =  below background.
